# Harnessing Microbiome, Bacterial Extracellular Vesicle, and Artificial Intelligence for Polycystic Ovary Syndrome Diagnosis and Management

**DOI:** 10.3390/biom15060834

**Published:** 2025-06-07

**Authors:** Bhawna Kushawaha, Tial T. Rem, Emanuele Pelosi

**Affiliations:** Department of Biochemistry and Molecular Biology, Indiana University, Indianapolis, IN 46202, USA; bkushaw@iu.edu (B.K.); trem@iu.edu (T.T.R.)

**Keywords:** polycystic ovary syndrome (PCOS), artificial intelligence (AI), microbiome, extracellular vesicles (EV), bacterial extracellular vesicles (BEV), ovary, AI in PCOS diagnostic

## Abstract

Polycystic ovary syndrome (PCOS) affects 6–19% of reproductive-age women worldwide, yet diagnosis remains challenging due to heterogeneous presentations and symptoms overlapping with other endocrine disorders. Recent studies have shown that gut dysbiosis plays a significant role in PCOS pathophysiology, with bacterial extracellular vesicles (BEVs) functioning as critical mediators of the gut–ovary axis. BEVs carry distinct cargos in PCOS patients—including specific miRNAs and inflammatory proteins—and show promise for both diagnostic and therapeutic applications. Artificial intelligence (AI) is emerging as a promising significant tool in PCOS research due to improved diagnostic accuracy and the capability to analyze complex datasets combining microbiome, BEV, and clinical parameters. These integrated approaches have the potential to better address PCOS multifactorial nature, enabling improved phenotypic classification and personalized treatment strategies. This review examines recent advances in the last 25 years in microbiome, BEV, and AI applications in PCOS research using PubMed, Web of Science, and Scopus databases. We explore the diagnostic potential of the AI-driven analysis of microbiome and BEV profiles, and address ethical considerations including data privacy and algorithmic bias. As these technologies continue to evolve, they hold increasing potential for the improvement of PCOS diagnosis and management, including the development of safer, more precise, and effective interventions.

## 1. Introduction

Polycystic ovary syndrome (PCOS) is characterized by a triad of symptoms including hyperandrogenism, ovulatory dysfunction, and polycystic ovarian morphology. PCOS is one of the most common endocrine disorders affecting women of reproductive age, with a global prevalence ranging from 6 to 19% using NIH criteria and from 8 to 13% using Rotterdam criteria [1,2,3,4]. The prevalence varies significantly by ethnicity and geographical region, with higher rates reported in South Asian (8–22%) and Middle Eastern populations (12–20%), while estimates are lower in East Asian populations (2.2–7.4%) (Table 1) [1,2,5]. The impacts of PCOS extend beyond reproductive health due to frequent association with metabolic conditions including insulin resistance and obesity [6]. Therefore, affected women face increased risks of type 2 diabetes, cardiovascular disease, and endometrial cancer. Despite high prevalence and significant health burden, the diagnosis of PCOS remains challenging due to heterogeneous presentations and the lack of a single definitive diagnostic test [7].

Recent advances in microbiome research have opened new avenues for understanding PCOS pathophysiology. Several studies have linked gut dysbiosis to various aspects of PCOS, including impaired insulin sensitivity, hyperandrogenism, and chronic low-grade inflammation [19]. Several studies have found that, compared to healthy controls, PCOS patients exhibit reduced α-diversity, which measures species richness and evenness within a single habitat or community [20,21,22]. However, other reports showed no significant differences [23,24]. While results on α-diversity seem conflicting, the evidence regarding β-diversity, which evaluates how species composition changes between different communities or habitats across environmental gradients, has been more consistent. Multiple studies showed that PCOS patients display distinct β-diversity patterns compared to healthy individuals [20,21,22,23,24,25,26].

**Figure 1:**
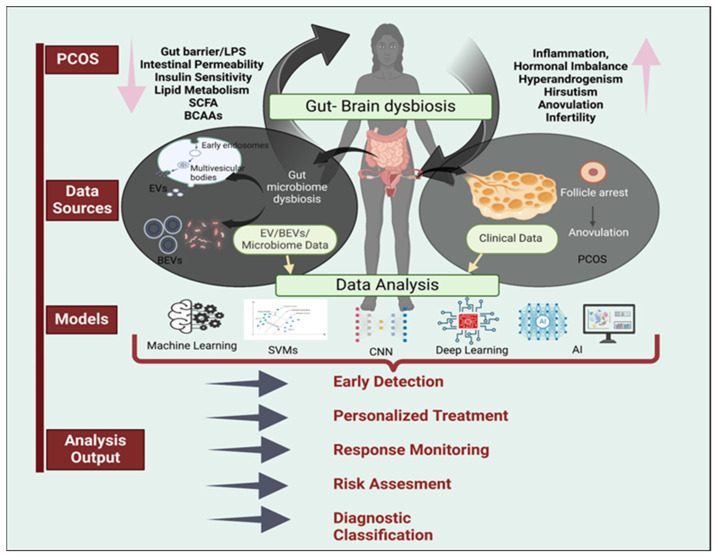
Integrated framework for the multidisciplinary diagnosis and management of PCOS: dysbiosis of the gut microbiome affects the metabolic and hormonal changes typical of PCOS pathophysiology. Data from multiple sources including microbiome, EVs, BEVs, and clinical parameters can be analyzed by several AI and machine learning tools including SVMs, CNNs, and deep learning. This integrated computational approach could improve the clinical management of PCOS by enabling earlier detection, personalized treatments, and better response monitoring, risk assessment, and diagnostic classification.

In parallel to microbiome research, the study of extracellular vehicles (EVs) or bacterial extracellular vesicles (BEVs) has emerged as a promising field in PCOS research. BEVs, nano-sized membrane vesicles released by bacteria, have gained attention as potential biomarkers of several reproductive conditions and important mediators of host–microbe interactions through their ability to deliver biomolecules to the host cells [27,28,29,30]. This interaction can in turn disrupt the delicate microenvironment within the reproductive system, therefore tipping the balance between health and disease. Joseph et al. studied the host–microbe communication in the cervicovaginal canal, and found that epithelial cells of the reproductive tract can internalize BEVs from several vaginal bacterial species, which were able to trigger a significant TLR2-specific immune [31]. Similarly, Oishi et al. reported significant differences in BEVs composition between women with and without endometriosis [16,32]. BEVs also seem to contribute to the development of PCOS by modulating inflammation, hormonal imbalances, and metabolic dysfunction. However, studies in this area are still few and further investigation is necessary to fully understand BEVs’ role in the pathogenesis of PCOS [33,34,35]. Yang et al. reported that, while BEV-associated inflammatory molecules can induce chronic inflammation in PCOS, BEVs may also improve metabolic disorders in PCOS by improving glucose and lipid metabolism and reducing adipose inflammation [29]. These findings evidence the complexity of the interactions occurring between bacteria and host cells, and suggest that the type of cargo delivered could affect how reproductive disorders arise and progress. Therefore, understanding the nature of microbiome–host communication could lead to important advancements in PCOS diagnosis and management [36,37,38,39,40,41]. An emerging area of clinical research involves harnessing artificial intelligence (AI) for the use of BEVs as diagnostic and therapeutic agents [42,43,44]. Due to the high computational capacity to analyze complex, high-dimensional data, AI holds significant potential for the improvement in clinical applications [45,46]. In the context of PCOS, AI could potentially integrate microbiome and BEV data with PCOS clinical parameters to develop more accurate and personalized diagnostic tools [44,47,48,49,50,51,52]. Although AI represents a promising opportunity to enhance our understanding of PCOS, it also raises important ethical considerations that need to be addressed, including privacy issues, social disparities, and access to care [45,53,54,55,56]. In this review, we explore the potential of integrating microbiome, BEVs, and AI to improve diagnostic and therapeutic strategies for PCOS. We examine current clinical approaches and limitations, recent findings on the role of microbiome and BEVs in PCOS pathophysiology, and potential applications of machine learning (ML) and AI (Figure 1). Furthermore, we address the ethical challenges of implementing these emerging and promising technologies in clinical practice.

## 2. Methods

We performed an exhaustive search of the literature from year 2000 to 2025 using PubMed, Web of Science, and Scopus databases, with additional articles identified through the reference list screening of included studies. The primary search terms included polycystic ovary syndrome OR PCOS in combination with (“artificial intelligence” OR “machine learning” OR “deep learning”) and (microbiome OR microbiota OR “gut flora”) AND (“extracellular vesicles” OR “bacterial extracellular vesicles”), PCOS biomarkers, EV microRNA in PCOS, Ovarian EVs in PCOS, and EV isolation techniques. Inclusion criteria encompassed (1) original research articles published in peer-reviewed journals; (2) studies focusing on PCOS diagnosis or management using AI, microbiome analysis, and/or BEV profiling; (3) human studies or in vivo animal models; and (4) articles published in English. Studies were excluded if they were (1) review articles, case reports, or conference abstracts; (2) studies not directly related to PCOS or the use of AI, microbiome, or BEV applications; (3) articles without full-text availability; and (4) studies with inadequate methodological descriptions or insufficient data reporting. Images were created in BioRender. Kushawaha, B. (2025) https://BioRender.com/3htq4d3.

## 3. Current Diagnostic Approaches for PCOS and Limitations

The diagnosis of PCOS has evolved significantly over the years. Currently, three are the main diagnostic frameworks being used worldwide (Table 2). The original NIH criteria [9,57] focused on the clinical manifestations of androgen excess and ovulatory dysfunction, providing good specificity but potentially missing broader phenotypic presentations. These criteria established PCOS as primarily a reproductive–endocrine disorder but failed to recognize significant metabolic components that are integral to its pathophysiology [11,58]. The subsequent Rotterdam criteria [4] incorporated ovarian morphology as a key feature, creating four distinct PCOS phenotypes with varying metabolic and reproductive profiles. These criteria required two of three features for a positive diagnosis: oligo-anovulation, clinical and/or biochemical hyperandrogenism, and polycystic ovarian morphology [4]. While this expanded definition increased diagnostic sensitivity, it introduced significant heterogeneity raising concerns regarding whether all identified phenotypes represented the same underlying disorder [59]. The Rotterdam criteria also suffer from practical implementation challenges, with studies demonstrating problematic inter-observer variability in ultrasound assessments and scoring of hirsutism [60,61,62,63,64]. The Androgen Excess Society (AES) criteria attempted to refocus the diagnosis on hyperandrogenism as the central feature while still considering ovarian dysfunction [9,18]. This approach offered advantages in identifying women at higher metabolic risk, but its clinical implementation poses challenges due to technical inadequacies in androgen measurement. Standard laboratory assays for testosterone demonstrated precision and reliability issues at the typical lower concentrations of women, with direct immunoassays being particularly unreliable [65]. Even with advanced approaches such as mass spectrometry, establishing universal thresholds for hyperandrogenemia remains problematic due to significant ethnic and age-related variations in androgen levels [66]. The clinical assessment of hyperandrogenism through hirsutism scoring also demonstrates poor standardization and high observer dependency [67]. A fundamental limitation across all diagnostic approaches is their failure to integrate metabolic parameters, despite evidence that insulin resistance affects 65–70% of PCOS patients and plays a crucial pathophysiological role [12,68]. A large-scale study involving 1212 patients found that the significant prevalence of insulin resistance occurs independently of obesity status, yet insulin resistance is not part of the diagnostic criteria, potentially leading to the underdiagnosis of metabolic complications [11].

The existing criteria also apply the same value thresholds across diverse populations despite mounting evidence of significant ethnic variations in hirsutism presentation, androgen levels, and follicle morphology [69,70]. Additionally, the broad nature of the Rotterdam criteria has led to concerns about overdiagnosis. Reports of PCOS prevalence range from 6% with the NIH criteria to 21% with the Rotterdam criteria [1]. This variability highlights the need for improved diagnostic methods. In addition, the Rotterdam approach, while widely adopted, demonstrates limited sensitivity (75%) despite high specificity (99%) when using the 12-follicle threshold for polycystic ovarian morphology [64,68,71]. A comparison between the updated 2018 guidelines and traditional Rotterdam criteria revealed that only 76% of women diagnosed via Rotterdam criteria met the newer guidelines [17,72].

Emerging new diagnostic approaches using microbiome and BEVs analyses have shown promising diagnostic capabilities (Table 2). Systematic reviews and research reported consistent microbial signatures in PCOS patients across different populations [16,22,25,38,40,63,73]. In addition, artificial intelligence-based diagnostic models have achieved remarkable accuracy, sensitivity, and specificity [43,74]. A comprehensive NIH systematic review spanning 25 years of research (1997–2022) confirmed that AI/ML techniques can effectively detect PCOS, potentially addressing the significant burden of under- and mis-diagnosed cases [43]. Overall, these findings suggest that integrating microbiome, AI, and BEV could significantly enhance PCOS diagnosis, therefore addressing the fundamental limitations of traditional criteria while providing more precise phenotypic stratification and personalized treatment pathways.

**Table 2:** Traditional and emerging diagnostic approaches for PCOS.

Diagnostic Approach	Key Biomarkers	Specific Values/Thresholds	Strengths	Limitations	Performance	References
**Traditional Approaches**						
NIH criteria (1992)	Hyperandrogenism: Elevated circulating androgens above 95th percentile of healthy controls OR clinical signs (hirsutism, acne, alopecia). Measured via total testosterone, free testosterone. Ovulatory dysfunction: Irregular or absent ovulation with menstrual irregularity, assessed through menstrual cycle patterns and ovulation markers.	Total testosterone: >88 ng/dL (>2.4 nmol/L); Free testosterone: >0.75 ng/dL; Oligomenorrhea: ≤8 cycles/year; Cycle length: >35 days or <21 days	High specificity (100%); Focus on reproductive-endocrine disorder components; Well-defined androgen thresholds	Fails to recognize metabolic components; Narrower phenotypic presentation; Limited sensitivity	Sensitivity: 60% Specificity: 100%	[57,75]
Rotterdam criteria (2003/updated 2023)	Oligo/anovulation: ≤8 menstrual cycles per year, assessed through cycle frequency and ovulation markers. Clinical hyperandrogenism: Hirsutism, acne, or androgenic alopecia measured by Ferriman–Gallwey score. Biochemical hyperandrogenism: Elevated testosterone or androstenedione levels. Polycystic ovaries: Increased follicle number (≥20) or ovarian volume (≥10 mL) on ultrasound using modern technology, assessed via follicle count and ovarian volume measurements.	Oligomenorrhea: ≤8 cycles/year; LH/FSH ratio: Often >2:1, Ferriman-Gallwey score: ≥8 (varies by ethnicity); Total testosterone: Variable by assay method, Updated criteria (2023); Follicle count: ≥20 per ovary (8 MHz transducer); Ovarian volume: ≥10 mL (either ovary), Previous: ≥12 follicles per ovary	Widely adopted in clinical practice; Updated follicle thresholds reflect improved imaging technology	Original 12-follicle threshold now considered too low; Inter-observer variability in ultrasound assessment; No incorporation of metabolic parameters	Original criteria: Sensitivity: 75% Specificity: 99% Updated follicle threshold reduces false positives	[4,76,77,78,79]
AE-PCOS Society criteria	Hyperandrogenism: Central diagnostic feature that must be present either clinically or biochemically, assessed through clinical manifestations and biochemical markers. Ovarian dysfunction: Either oligo/anovulation OR polycystic ovaries on ultrasound, evaluated via menstrual irregularity and polycystic ovaries assessment.	Clinical hyperandrogenism: Present; Biochemical hyperandrogenism: Method-dependent thresholds; Oligo/anovulation: Present; Polycystic ovarian morphology: As per updated criteria	Emphasizes hyperandrogenism as core feature; Better identification of women with metabolic risks	More restrictive than Rotterdam; Excludes some milder phenotypes; Implementation challenges	Performance metrics not extensively validated in large studies	[9,18]
**Emerging Approaches**						
Microbiome Analysis	Gut dysbiosis: Altered microbiota composition characterized by reduced diversity and specific bacterial imbalances associated with metabolic dysfunction. Assessed via Firmicutes/Bacteroidetes ratio, specific bacterial genera (Escherichia-Shigella, Proteobacteria), alpha diversity measures, and beta diversity patterns. Microbiome–PCOS axis: Gut bacteria influence host metabolism, inflammation, and hormone regulation.	PCOS vs. Controls: Decreased Firmicutes/Bacteroidetes ratio; Increased Proteobacteria abundance; Increased Escherichia-Shigella: Variable but often elevated; Decreased overall alpha diversity Note: Specific thresholds vary significantly between studies and populations	Insights into pathogenesis; Potential therapeutic targets through microbiome modulation; Non-invasive sample collection	High inter-individual variability; Lack of standardized collection/analysis methods; Confounding by diet and lifestyle; Limited clinical validation	Machine learning classification accuracy varies widely; No consistent diagnostic thresholds established	[20,23,80,81,82]
Bacterial Extracellular Vesicles (BEVs)	EV dysregulation: Altered bacterial and cellular extracellular vesicle cargo reflecting systemic inflammation and metabolic dysfunction. Measured via various miRNA species, protein cargo markers, and cytokine profiles in EVs. Intercellular communication: EVs carry regulatory molecules between cells and tissues, serving as biomarkers for disease state.	Research-stage biomarkers: miRNA expression patterns: Study-dependent fold changes; EV concentration: Often elevated in PCOS; Inflammatory protein cargo: Variable across studies Note: Specific diagnostic thresholds not established	Potential for multi-parameter biomarker panels; Reflects systemic pathophysiology; Stable in circulation	Primarily used in base research; Standardization of isolation methods needed; Limited clinical validation studies; High technical complexity	Research-stage metrics: Various AUC values reported (0.8–0.95) in preliminary studies	[83,84,85]
Artificial Intelligence—Clinical Data	Machine learning classification: Algorithmic integration of multiple clinical parameters to generate diagnostic probability scores using various ML techniques (SVM, Random Forest, etc.). Input features include clinical features, laboratory values (BMI, testosterone levels, cycle regularity, LH/FSH ratios), and anthropometric measures. Output includes probability scores and classification decisions.	Algorithm performance varies: Feature combinations: Study-dependent; Probability thresholds: Typically > 0.5 for positive classification; Cross-validation: k-fold approaches Common features: BMI, testosterone levels, cycle regularity, LH/FSH ratios	High diagnostic accuracy; Integration of multiple data types; Objective decision making; Potential for clinical decision support	Need for large, diverse training datasets; Potential algorithmic bias; Model interpretability challenges; Validation across populations needed	Overall Performance: AUC: 73–100% Accuracy: 89–100% Sensitivity: 41–100% Specificity: 75–100% Standardized criteria studies: AUC: 80–100% Accuracy: 89–100%	[43,44,86,87]
Deep Learning—Ultrasound Image Analysis	Automated image analysis: Computer vision algorithms for objective ultrasound interpretation with automated feature extraction and pattern recognition. CNN-based features include automated follicle detection, ovarian morphology analysis, and texture and pattern recognition. Deep feature learning: CNNs learn hierarchical representations directly from image data without manual feature engineering, processing pixel-level analysis and feature extraction.	Technical specifications: Input image resolution: Typically 224 × 224 pixels; Follicle detection: Automated counting and sizing; CNN architectures: VGG16, ResNet, Inception V3, custom designs performance thresholds: Classification confidence: >0.5 probability; Image quality requirements: Variable by study	Reduced inter-observer variability; Objective measurements; Potential for real-time diagnosis; Automated follicle counting; Reduced dependency on operator expertise	Computational requirements; Need for large, annotated datasets; Model generalizability across different ultrasound systems; Black box interpretability	Individual studies: VGG16+XGBoost: 99.89% accuracy (Suha & Islam, 2022); Various CNN models: 82.6–99% accuracy; Sensitivity: 85–100%; Specificity: 80–94%; Precision: 82.6–97%	[88,89,90,91,92]
Integrated Multi-omics AI	Precision medicine approach: Integration of genetic, molecular, clinical, and imaging data for comprehensive phenotyping and personalized risk assessment. Input data includes genomic variants, clinical phenotypes, laboratory biomarkers, and imaging data. Systems biology: Understanding PCOS as a complex multi-system disorder with individualized presentations through multi-modal integration and pathway analysis.	Complex feature integration: SNP risk scores: Population-dependent; Multi-omics data fusion: Study-specific approaches; Ensemble methods: Combined algorithm outputs; Personalized risk stratification: Individual-based thresholds	Comprehensive molecular profiling; Individual risk stratification; Potential for personalized treatment; Integration of diverse data types	High cost and complexity; Data privacy concerns; Limited clinical accessibility; Standardization challenges; Requires specialized infrastructure	Research-stage metrics: Limited large-scale validation studies available; Promising preliminary results in small cohorts	[93]

Abbreviations: ASRM—American Society for Reproductive Medicine, AE-PCOS—Androgen Excess and PCOS Society ML—Machine Learning, SVM—Support Vector Machine, CNN—Convolutional Neural Networks, VGG16—Visual Geometry Group 16-layer network, ResNet—Residual Networks, XGBoost—Extreme Gradient Boosting, AUC—Area Under the Curve, ng/dL—nanograms per deciliter, nmol/L—nanomoles per liter, MHz—megahertz, mL—milliliters.

## 4. Microbiome Analysis in PCOS

In 2012, the dysbiosis of gut microbiota (DOGMA) hypothesis suggested that, following an imbalance in intestinal flora, an increase in intestinal permeability could cause the leakage of lipopolysaccharide (LPS) into the systemic circulation leading to inflammatory response and insulin resistant [19]. Since then, PCOS has increasingly been linked to changes in the gut microbiome. Recently, Yang et al. reanalyzed raw sequencing data from 14 publications involving 948 individuals between 2010 and 2024, revealing distinct microbial signatures in PCOS patients despite finding no significant differences in α-diversity compared to healthy controls. The study identified increased abundances of *Fusobacterium*, *Ruminococcus gnavus* group, and *Escherichia-Shigella*, while noting decreases in *Dysosmobacter*, *Schaedlerella*, *Merdimonas*, *Clostridiisalibacter*, and *Flintibacter* in PCOS patients. Importantly, the analysis uncovered distinct microbial profiles based on testosterone levels, and identified a set of eight genera differentiating high-testosterone from low-testosterone PCOS patients with an AUC of 0.95 [29]. These results suggested that the microbiome has potential diagnostic applicability and provided insights for targeted therapeutic strategies [29]. Furthermore, Insenser et al. studied 15 PCOS patients and 16 non-hyperandrogenic control women, and found significant associations between gut microbiome composition and sex hormones, with bacterial α-diversity showing positive correlations with total testosterone (*p* = 0.027) and the ratio of free testosterone to free estradiol (*p* = 0.007), while negatively correlating with total estradiol (*p* = 0.041). PCOS patients showed specific alterations in gut microbiota, particularly an increased abundance of *Catenibacterium* and *Kandleria* genera. The abundance of the *Candidatus saccharibacteria* phylum was significantly higher in obese patients, and *Kandleria* showed a positive correlation with circulating androstenedione concentrations. In obese PCOS patients, β-diversity was particularly reduced, suggesting a complex interaction between obesity, hormonal status, and gut microbiome [21]. Similarly, the analysis of the fecal microbiome from 24 women with PCOS and 19 healthy controls revealed significant differences between these two groups [20]. The authors observed that PCOS patients had a lower relative abundance of three bacterial taxa, the phylum Tenericutes, the order ML615J-28 (belonging to the phylum Tenericutes), and the family S24-7 (belonging to the phylum Bacteroidetes). These changes correlated with reproductive parameters and elevated testosterone levels in PCOS. The significant reduction in gut microbiome diversity observed in PCOS patients (15% lower Faith’s phylogenetic diversity, *p* < 0.03) suggests a less complex and potentially unstable microbial ecosystem. The authors also found increased serum zonulin (*p* = 0.006), higher diamine oxidase (*p* = 0.044), and increased lymphocytes (*p* = 0.001) levels in PCOS compared to controls. These data show a potential mechanism linking gut dysbiosis to PCOS, proposing possible biomarkers for microbiome-based diagnostics [20]. Building upon these findings, Torres et al. conducted a larger study involving 73 women with PCOS and 48 healthy controls [22]. The study found a strong association between microbiome changes and hyperandrogenism, with both total testosterone levels and hirsutism scores negatively correlating with α-diversity. The analysis of β-diversity showed the significant effects of hyperandrogenism on microbial community composition (*p* = 0.0009). Random forest analysis identified specific bacterial changes, with an increased abundance of *Porphyromonas* spp., *Bacteroides coprophilus*, *Blautia* spp., and *Faecalibacterium prausnitzii*, and decreased levels of *Anaerococcus*, *Odoribacter*, *Roseburia*, and *Ruminococcus bromii* in PCOS patients. Interestingly, while hyperandrogenism strongly correlated with microbiome changes, body mass index (BMI), and homeostatic model assessment for insulin resistance (HOMA-IR) did not, suggesting that androgens may play a crucial role in shaping the gut microbiome in PCOS patients. Zhang et al. found that *Faecalibacterium prausnitzii* and *Bifidobacterium* spp. were reduced in PCOS patients and correlated positively with levels of short-chain fatty acid (SCFA), bacterial metabolites produced during fiber digestion [24]. Bacteria from the Bacteroidaceae family were elevated in PCOS patients and associated with insulin resistance and inflammation, whereas Prevotellaceae were decreased with a negative correlation with testosterone levels [24]. Liu et al. identified significant differences in gut microbial composition among PCOS patients, characterized by reduced *α-diversity* and notable shifts in bacterial populations. Specifically, they observed increases in LPS-producing bacteria, particularly from genera *Bacteroides* and *Escherichia/Shigella*, alongside decreases in spore-forming species, including genera *Akkermansia* and *Ruminococcaceae*. The study identified 23 bacterial co-abundance groups, which showed significant correlations with clinical parameters. The dysbiosis pattern demonstrated strong associations with multiple metabolic and endocrine markers, including obesity, inflammatory factors, insulin resistance, and hyperandrogenism. PCOS patients exhibited significantly decreased levels of brain–gut axis mediators, including serotonin, ghrelin, and peptide YY, which correlated negatively with waist circumference and testosterone levels [75]. Taken together, these findings show interesting associations between gut microbial dysbiosis and PCOS, suggesting a potential role in pathogenesis through complex interactions with metabolic and endocrine pathways.

Further evidence of a possible causative relationship comes from transplantation experiments of intestinal bacteria from PCOS patients into mice [23]. Following transplantation, recipient mice developed PCOS-like symptoms, accompanied by decreased levels of bile acids glycodeoxycholic acid (GDCA) and tauroursodeoxycholic acid (TUDCA), and intestinal IL-22. Notably, the treatment of PCOS mice with either GDCA or IL-22 led to significant improvements in hormone regulation, insulin sensitivity, ovarian morphology, and fertility. Reduced abundance in *Roseburia* was a common finding in several studies, and could provide insights into molecular mechanisms [76,77]. This genus is a major producer of the SCFA butyrate, which has been shown to improve insulin sensitivity through processes including mitochondrial fatty acid oxidation and a reduction in inflammatory cytokines by inhibiting the NF-κB pathway [78,79]. This is particularly relevant to PCOS, where both insulin resistance and chronic inflammation contribute to ovarian dysfunction and hormonal imbalances [21]. Liu et al. reported decreased butyrate concentrations in the serum of obese PCOS patients compared to controls, and in vitro experiments showed that the addition of butyric acid correlated with changes in the m6A epigenetic marker and a decreased production of inflammatory factors IL-6 and TNF-α through the *METTL3*-mediated regulation of *FOSL2* [80]. The dysregulation of these inflammatory mediators created a feedback loop further exacerbating the insulin resistance. [80]. Furthermore, the supplementation of butyrate in PCOS rats ameliorated both metabolic and endocrine disruptions by mitigating hyperandrogenism, insulin resistance, and inflammatory markers (NF-kB, TNF-α), while enhancing mitochondrial function through SIRT1-dependent pathways [81]. These studies provide a mechanistic link between the reduction in butyrate-producing bacteria and PCOS, while also highlighting a potential role of butyrate as therapeutic target [82]. The genus *Bacteroides* also seems to be involved in PCOS-associated gut dysbiosis, with several studies reporting significant changes in *Bacteroides* species abundance between PCOS patients and healthy controls [22,23,24,73,75,79]. The role of these bacteria seems to be mediated by three main mechanisms (Sonnenburg and Bäckhed 2016) [83]. First, *Bacteroides* species significantly impact bile acid metabolism through the modulation of FXR signaling pathways and the regulation of glucose homeostasis, which in turn affect IL-22 production [23]. Additionally, *Bacteroides* species produce branched-chain amino acids (BCAAs) that activate the mTOR signaling cascade and stimulate protein S6 kinase 1 (S6K1). This leads to the induction of insulin resistance through the activation of several downstream kinase pathways [84]. Lastly, the production of LPS contributes to metabolic endotoxemia and modulates systemic inflammation [85]. In a microbiome study of stool samples, *Bacteroides*, together with *Faecalibacterium* and *Bifidobacterium*, were identified by random forest classifier among the top 10 bacterial genera able to discriminate PCOS from controls [73]. The model achieved 87.5% accuracy (95% CI: 84.2–90.8%), 89.3% sensitivity (95% CI: 85.6–93.0%), 85.7% specificity (95% CI: 81.4–90.0%), and 0.93 AUC-ROC (95% CI: 0.90–0.96). Unlike *Bacteroides*, *Faecalibacterium* and *Bifidobacterium* showed inverse correlations with PCOS severity and a protective anti-inflammatory effects by enhancing butyrate production and strengthening intestinal barrier integrity [86]. The further understanding of the complex interplay between bacterial species and their metabolic products with the host will offer valuable insights into disease progression and the use of the gut microbiome as therapeutic target.

## 5. EV and BEVs Analysis in PCOS

In recent years, EVs have emerged as critical bioparticles with promising diagnostic applicability through the analysis of their cargo, which faithfully reflects the biology of the cells of origin. Recently, Duval et al. presented a detailed review on follicular fluid EVs (FFEVs) in PCOS patients, and identified specific miRNAs, such as *miR-379* and *miR-200*, as potential markers of reproductive dysfunction [87]. Further, Park et al. reported how mesenchymal stem cell-derived EVs (MSC-EVs) could restore fertility in a PCOS mice model by downregulating the expression of Cyp17a1 and Dennd1 []. Furthermore, *miR-323-3p* derived from MSC-EVs, was found to reduce granulosa cell apoptosis, improve follicle development, and regulate inflammation in PCOS [88]. Hyperandrogenism promotes a proinflammatory environment in follicular macrophages, shifting them towards the M1 state and increasing the M1/M2 ratio due to a decline in anti-inflammatory M2 macrophages [89]. In PCOS, Salehi et al. reported that *miR-379-5p* carried by granulosa cell-derived EVs led to an inhibition of M2 macrophage polarization and an elevation of the M1/M2 ratio [90]. This also resulted in the enhanced secretion of the proinflammatory cytokine galectin-3, which in turn suppressed granulosa cell proliferation in a follicle-stage-dependent manner [90]. In addition, by comparing small EVs (sEVs) in the follicular fluid of PCOS and matched control patients, 26 miRNAs were identified and predicted to regulate specific target genes predominantly affecting the MAPK and PI3K-Akt pathways. Seven miRNAs showed a significantly elevated expression in PCOS patients, primarily affecting folliculogenesis and oocyte maturation [91,92]. A separate study identified *miR-27a-5p* as being differentially expressed in PCOS patients compared to controls [93]. A meta-analysis by Deswal and Dang evaluated 79 miRNAs across 21 studies, identifying *miR-29a-5p*, *miR-320*, and *miR-93* as consistently altered in PCOS, and ROC analysis established *miR-29a-5p* as a superior diagnostic marker (AUC = 0.95) [94]. An analysis of the follicular fluids of PCOS patients showed *miR-143-3p* and *miR-155-5p* as potential factors in PCOS pathogenesis [95]. These miRNAs were shown to modulate glycolysis through hexokinase 2, therefore affecting ATP production and cell survival. In addition, reduced glycolytic activity was shown to accelerate KGN cell apoptosis, further contributing to follicular dysplasia [95]. Udesen et al. identified three miRNAs with strong diagnostic potential, *miR-139-5p* (AUC = 0.857), *miR-376a-3p* (AUC = 0.838), and *miR-28-3p* (AUC = 0.807), outperforming traditional markers [96]. Wang et al. [97] reported an elevated *miR-27a-3p* expression in PCOS mouse ovaries, affecting granulosa cell function through the targeting of *CREB1* and the steroidogenic pathways. Further research by the same group in 21 PCOS and 12 controls revealed a role for *miR-27a-3p* in insulin resistance through *STAT1/STAT3* mediation and *SMAD5* regulation [97,98].

In addition to EVs being released by host cells, BEVs are gaining traction as biomarkers of PCOS [34,37,99]. Following metformin treatment, Hu et al. reported a significant increase in Lactobacillales-derived EVs (0.51% to 6.87%), particularly from *Streptococcus salivarius* (0.03% to 4.33%) in the plasma of PCOS-IR patients [100]. Additionally, the authors observed post-treatment shifts in bacteria populations, with changes in *Sphingobacterium hotanense* and *Bradyrhizobium* levels, alongside a transition from Bacteroidetes phylum to Firmicutes dominance. In addition to miRNA, it was shown that BEVs mediate cellular communication through the transfer of proteins, particularly TNF-α and IL-6, which activate NF-κB pathway-mediated inflammatory cascades [101,102,103]. The resulting inflammation impaired insulin signaling through IRS-1 serine phosphorylation [104].

Alongside disease diagnosis, EVs show promise as delivery systems for therapeutic applications [36,105]. BEVs could be engineered for the delivery of drugs or biomolecules through biofilm formation and nucleation with extracellular DNAs [106,107,108,109]. As androgens are involved in several aspects of PCOS pathology including follicular arrest, insulin resistance, and inflammation, the androgen receptor may represent a potential therapeutic target through AR-siRNA-loaded BEVs [109]. By engineering EVs from the adipose mesenchymal stem cells (AMSCs) to carry *miR-21–5p*, Cao et al. showed improved ovarian function and insulin sensitivity in PCOS mice models by activating the IRS1/AKT pathway and increasing hepatic metabolism [110].

## 6. Artificial Intelligence and Machine Learning in Medical Diagnostics of PCOS

Artificial intelligence has shown promising results for the diagnosis and management of PCOS [43,44,111,112] (Table 3). While microbiome and BEV analyses yield valuable insights, they also generate vast and complex datasets that pose challenges for interpretation using traditional computational methods.

AI, particularly machine learning, has shown remarkable potential in analyzing complex medical data for disease detection and classification [136]. Zhang et al. achieved a diagnostic accuracy of 92.0% (CI: 88.9–95.1%), with 93.0% sensitivity, 91.0% specificity, and 0.96 AUC-ROC when using machine learning to integrate gut microbiome, BEV-associated miRNAs, and clinical parameters compared to the use of individual approaches [137,138]. The use of multi-level investigations is particularly attractive because it could better address the complex interplay of the multiple molecular mechanisms regulating PCOS pathogenesis including (1) metabolic changes induced by microbiome-derived signals, (2) endocrine disruptions via BEV-associated miRNAs, and (3) systemic inflammation assessed by clinical evaluation [137]. In a study of 721 women (388 PCOS patients and 333 controls), Lv et al. developed a new deep learning approach for the detection of PCOS through non-invasive eye imaging. The method employed a three-stage strategy: first, segmentation of full-eye images by enhanced U-Net architecture; second, extraction of deep features from the segmented scleral images by ResNet architecture; finally, diagnostic classification by multi-instance learning model. The approach showed high performance with an AUC of 0.979 and classification accuracy of 0.929 [127]. These results suggest the potential of deep learning-based analyses for the diagnosis of PCOS and could prove particularly valuable in early detection compared to traditional diagnostic methods.

Several AI analytical methods have shown promise in PCOS research, offering new avenues for more accurate and efficient diagnostic procedures [139]. These include the methods given below.

### 6.1. Random Forest

Random forest models could become reliable tools for PCOS screening [124]. In a dataset of 541 patients, a random forest classifier was trained on multiple clinical parameters including hormonal profiles, ultrasound findings, and metabolic markers [113]. The feature importance analysis revealed that the most significant predictors of PCOS included AMH, testosterone, and follicle count. The model achieved high diagnostic accuracy with performance metrics showing 98% accuracy, 97% precision, 98% recall, and 98% F1-score. Similarly, Tiwari et al. [140] reported that random forest outperformed other machine learning algorithms, showing an accuracy of 93.25%, and improved performance in identifying complex patterns within the clinical data.

### 6.2. Support Vector Machines (SVMs)

Song et al. employed SVMs to classify PCOS patients based on serum metabolomic profiles [141]. Using gas chromatography–mass spectrometry, they identified 30 metabolites that were significantly different between PCOS and control groups (*p* < 0.05). The AUC-ROC of the SVM model was 0.935 (95% CI: 0.898–0.972), demonstrating high diagnostic accuracy, and the potential of metabolomic profiling combined with machine learning in PCOS diagnosis. Furthermore, Kodipalli and Devi combined fuzzy logic and machine learning to develop the Fuzzy tTechnique for Order of Preference by Similarity to Ideal Solution (Fuzzy-TOPSIS) method for the early detection of PCOS and associated mental health issues. Fuzzy TOPSIS achieved superior accuracy of 98.20% compared to SVM’s 94.01%. The methodology incorporated linguistic responses typical of clinical settings, showing applicability in typical situations when patients face uncertainty describing their symptoms [114].

### 6.3. Deep Learning for Image Analysis

(a) Convolutional neural networks (CNNs): The CNN model, based on the ResNet-50 architecture, achieved 92.3% accuracy (95% CI: 90.1–94.5%), 91.4% sensitivity (95% CI: 88.7–94.1%), and 93.1% specificity (95% CI: 90.6–5.6%) in identifying polycystic ovary morphology, significantly outperforming traditional manual assessments [108,120,124,132,142,143,144,145,146,147,148]. In a two-phase approach, Gülhan et al. optimized follicle detection in ultrasound images through several preprocessing methods, followed by a CNN-based classification of ovarian images. The technique discriminated between normal and PCOS images, with accuracies of 65.81% for raw images and 77.81% for preprocessed images [122]. Similarly, Sumathi et al. used CNN-based image processing to classify ovarian cysts, achieving 85% accuracy [121]. Overall, these studies suggest that CNN-based approaches, particularly when combined with optimized preprocessing methods, offer promising potential for automated PCOS detection through ultrasound image analysis.
(b) Advanced CNN architectures: Suha and Islam combined the CNN architecture for feature extraction and a stacking ensemble method for classification [123]. Compared to existing machine learning methods, this approach improved the accuracy and reduced training time, resulting in 99.89% classification accuracy. Furthermore, Garzia et al. investigated predictors of metformin treatment effectiveness in PCOS patients using artificial neural networks (ANNs), specifically focusing on weight loss and androgen level reduction outcomes. Using Auto-CM, a fourth-generation ANN, the authors developed semantic connectivity maps (SCMs) to correlate baseline clinical characteristics with treatment outcomes. The ANN analysis revealed that patients with oligo-amenorrhea and hyperandrogenemia at baseline were most likely to respond positively to metformin treatment, whereas lower baseline testosterone levels was a significant predictor of treatment discontinuation [131].

### 6.4. Integrated Approaches

The use of machine learning models to analyze multiple different datasets including laboratory, clinical, and image data may pave a promising path towards more rapid, accurate, and potentially cost-effective PCOS diagnostic procedures. Kermanshahchi et al. developed a machine learning model for identifying PCOS from pelvic ultrasound images, based on detecting multiple small follicles and increased ovarian volume, both key indicators of PCOS [130]. Although the model showed 100% sensitivity and specificity in distinguishing PCOS, the authors acknowledge the need for further validation studies. They also suggest that future work should incorporate additional diagnostic factors such as physical exam findings and laboratory values to create a more comprehensive and robust approach. Shanmugavadivel et al. used deep learning to analyze both clinical data and ultrasound images [149]. The study showed that a SVM model achieved the highest accuracy for clinical data, and a VGG16 transfer learning approach outperformed other models for ultrasound image analysis. Although promising, more studies are needed to fully understand their applicability. Additionally, the validation of these models in larger, more diverse patient populations and across independent analyses will be critical milestones in the future.

## 7. Combining AI with Microbiome and BEVs Analysis for PCOS Diagnosis

Research into the use of AI for the application of microbiome and BEVs profiles to PCOS diagnosis could represent a significant advancement in the field, while also providing insights into molecular mechanisms and potential therapeutic targets.

### 7.1. Data Collection and Preprocessing

The development of standardized sample collection methods is critical for the efficient use of AI-based microbiome and BEV analyses in PCOS research. To ensure comparability across studies, Knight et al. recommended using the Earth Microbiome Project protocols for sample collection and processing [150]. For BEV isolation, ultracentrifugation combined with density gradient purification is considered the gold standard, but new techniques are constantly being developed including tangential flow filtration, and may lead to improved purity and yields [151].

### 7.2. Feature Selection and Model Development

Lindheim et al. collected stool samples from 24 PCOS patients and 19 healthy controls. After quality filtering and chimera removal, 1237 operational taxonomic units (OTUs) were identified [20,25]. Random forest algorithms were used for feature selection and classification. The study identified 12 bacterial genera as potential biomarkers, with *Bacteroides*, *Ruminococcus*, and *Faecalibacterium* showing the highest importance (mean decrease in Gini index > 2.0). The model achieved an AUC-ROC of 0.79 (95% CI: 0.69–0.92) in distinguishing PCOS patients from controls. Liu et al. developed a machine learning model to analyze gut microbiome and clinical data from 106 PCOS patients and 102 healthy controls. The authors also employed a random forest algorithm for classification, achieving an accuracy of 85% (95% CI: 81–89%), sensitivity of 87% (95% CI: 82–92%), and specificity of 83% (95% CI: 78–88%) [75]. Importantly, the integration of microbiome data significantly improved the model’s performance compared to clinical data alone (AUC-ROC: 0.91 vs. 0.83, *p* < 0.01).

### 7.3. AI-Enabled PCOS Subtyping

The heterogeneity of PCOS presents a significant challenge in clinical diagnosis and management. AI offers the potential to identify distinct PCOS phenotypes based on microbiome and BEV profiles. The use of unsupervised machine learning with k-means clustering and principal component analysis allowed the identification of four distinct PCOS subtypes: (1) mild, characterized by mild hyperandrogenism and normal metabolic parameters; (2) reproductive, showing severe hyperandrogenism and oligo/anovulation, but a normal metabolic profile; (3) metabolic, featuring insulin resistance, obesity, and moderate hyperandrogenism; and (4) severe, characterized by severe hyperandrogenism, metabolic dysfunction, and the highest prevalence of polycystic ovary morphology [152]. While some studies did not incorporate microbiome data, they show the potential of AI in identifying clinically relevant PCOS subtypes, and the future inclusion of microbiome and BEV profiles could further refine this approach [27,44,117,126,139,142,149,152,153,154,155].

## 8. Towards Personalized Treatment of PCOS

Recent advancements in the AI analysis of microbiome and BEV data have opened new avenues for the personalized treatment of PCOS. Qi et al. conducted a randomized controlled trial to evaluate the efficacy of dietary interventions based on an AI-derived analysis of gut microbiome profiles [156]. The study included 86 women with PCOS, randomly assigned to either a personalized diet group (*n* = 43) or a standard diet group (*n* = 43). Following the identification of microbial signatures associated with treatment responses using a random forest algorithm, the personalized diet group experienced significantly greater improvements in insulin sensitivity (HOMA-IR decreased by 2.1 ± 0.5 vs. 1.2 ± 0.4, *p* < 0.001) and menstrual regularity (68% vs. 42%, *p* < 0.05) compared to the standard diet group. The AI model identified *Akkermansia muciniphila* and *Faecalibacterium prausnitzii* as key bacterial species associated with positive treatment outcomes. In addition, Fu et al. developed a machine learning model to predict responses to metformin treatment in PCOS patients [46,118]. Baseline clinical, hormonal, and metabolomic data were used to train a gradient boosting model to predict treatment response, defined as an improvement in menstrual regularity and a reduction in HOMA-IR. The model achieved an AUC-ROC of 0.83, and key predictive features included baseline insulin levels, BMI, and specific metabolites such as branched-chain amino acids.

Overall, AI is showing significant capability and versatility that may improve the early detection of PCOS phenotypes, provide deeper insights into the pathogenic molecular mechanisms, and even the predict success of personalized therapeutic strategies. However, the potential instability of microbiome populations over time represents a significant challenge for clinical translatability. Therefore, to fully validate AI programs and capture PCOS heterogeneity, the development of large-scale, longitudinal studies that employ multi-omics approaches, including genomics, metabolomics, and microbiomics becomes critical.

## 9. Ethical Challenges in AI and Microbiome-Based Approaches

While the potential benefits of AI-driven microbiome-informed approaches to PCOS diagnosis and treatment are significant, the application of these advanced technologies in healthcare also raises important ethical considerations.

### 9.1. Data Privacy and Security

The uniquely personal nature of microbiome data presents an unprecedented privacy risk, potentially revealing sensitive information far beyond the scope of PCOS diagnosis. Therefore, robust data protection, storage, and sharing policies need to be developed [54,56,157].

### 9.2. AI Bias

The development of AI models could perpetuate and amplify existing disparities [53]. The underrepresentation of minority populations in training datasets could lead to skewed diagnoses and treatments, potentially exacerbating health inequities in PCOS management. To mitigate these issues, proper data collection and regular bias audits need to be implemented while ensuring transparency in AI model development [158].

### 9.3. Regulatory Frameworks

The FDA Artificial Intelligence and Machine Learning Software as a Medical Device (AI/MLSaMD) action plan is an important first step for standardization and regulation, and similar frameworks should be implemented across other countries [159]. However, the dynamic nature of AI systems will likely present continuous new challenges, especially in healthcare. Therefore, adaptive regulatory protocols will be required to ensure rigor and patient safety [135].

## 10. Conclusions

The combination of microbiome analysis, BEV profiling, and AI represents a promising and innovative strategy for the diagnosis and management of PCOS. Several studies have revealed significant changes in gut microbiome composition in PCOS patients, while BEVs have emerged as potential biomarkers and mediators of PCOS pathophysiology. AI algorithms have shown improved accuracy in PCOS diagnosis using several data types, including clinical, metabolic, and imaging. These new technologies and approaches hold significant potential for the classification of PCOS subtypes, and the development of more targeted interventions. A key factor to advance our understanding of PCOS pathophysiology will be the continuous integration of multi-omics approaches including metabolomics, proteomics, and transcriptomics data. However, several ethical challenges exist including data privacy and AI bias. Addressing these issues while developing large-scale interdisciplinary studies will be crucial to ensure clinical translation and provide more effective interventions for women affected by this complex disorder.

## Figures and Tables

**Table 1:** PCOS epidemiology, clinical features, and diagnostic approaches.

Category	Description	Key Statistics/Features	References
Epidemiology	Global prevalence	6–19% of reproductive-age women worldwide using NIH criteria; 8–13% using Rotterdam criteria	[1,3,4,8]
	Ethnic variations	Higher rates in South Asian (8–22%) and Middle Eastern populations (12–20%); lower in East Asian populations (2.2–7.4%)	[1,5,9]
Clinical Features	Reproductive manifestations	Hyperandrogenism, ovulatory dysfunction, polycystic ovarian morphology	[4,6]
	Metabolic manifestations	Insulin resistance (65–70% of PCOS patients), obesity, increased risk of type 2 diabetes	[10,11,12]
	Other health risks	Cardiovascular disease, endometrial cancer	[6,11,13,14,15,16]
Traditional Diagnostic Approaches	NIH criteria (1992)	(1) Hyperandrogenism, (2) oligo/anovulation, (3) exclusion of other disorders	[3]
	Rotterdam criteria (2003)	Requires two of three features: (1) oligo/anovulation, (2) clinical/biochemical hyperandrogenism, (3) polycystic ovaries on ultrasound	[4]
	Androgen excess society criteria	(1) Hyperandrogenism, (2) ovarian dysfunction (oligo/anovulation and/or polycystic ovaries)	[17,18]

**Table 3:** AI/ML applications in PCOS diagnosis and management.

AI/ML Technique	Data Type and Sample Size	Validation Method	Feature Selection/Preprocessing	Performance	Key Findings	References
**Clinical Data Analysis**						
Random forest ensemble (multi-stack)	Clinical parameters (hormonal profiles, ultrasound findings, metabolic markers), *N* = 541	5-fold cross-validation	Mutual Information (MI) feature selection, SMOTEENN balancing	Accuracy: 98%, precision: 97%, recall: 98%, F1-score: 98%	Best performing model with explainable AI integration using SHAP, LIME	[113,114,115]
Random forest with ANN	Gene expression data (GEO database), *N* = 133 (76 PCOS, 57 controls)	Two training sets, two validation sets	12 key genes selected from 264 DEGs	AUC: 0.7273 (microarray), 0.6488 (RNA-seq)	Combined RF and neural network approach for gene biomarker identification	[116]
Hierarchical random forest ensemble	Clinical features with XAI, *N* = 541	8-fold cross-validation, 25 runs	TOMIM, TOPCA, OSSM feature selection methods	Accuracy: 99.31% (top 17 features), overall: 99.32%	Two-level ensemble with explainable AI using Shapash library	[117]
Support vector machines (SVMs)	Serum metabolomic profiles, metformin efficacy prediction, study-specific cohorts	Cross-validation	Metabolomic profiling	AUC-ROC: 0.935 (95% CI: 0.898–0.972)	Metabolomics-based prediction of treatment response	[118,119]
Fuzzy-TOPSIS + SVM	Clinical data with linguistic responses, study-specific	Not specified	Fuzzy logic preprocessing	Fuzzy-TOPSIS: 98.20%, SVM: 94.01%	Integration of fuzzy logic with traditional ML	[114]
**Image analysis**						
CNN (ResNet-50)	Ultrasound images, study-specific	Standard train/test split	Image preprocessing, augmentation	Accuracy: 92.3% (95% CI: 90.1–94.5%), Sensitivity: 91.4% (95% CI: 88.7–94.1%), Specificity: 93.1% (95% CI: 90.6–95.6%)	ResNet-50 architecture for ultrasound analysis	[120,121]
CNN (VGG16+XGBoost stacking)	Ultrasound images, *N* = 594 ovary USG images	Train/validation/test split	Transfer learning with VGG16, feature extraction	Accuracy: 99.89%, execution time optimized	Hybrid approach combining CNN and ensemble learning	[122,123]
CNN (various architectures)	Ultrasound images, variable by study	Train/test splits	Preprocessing: contrast enhancement, noise reduction	Raw images: 65.81%, preprocessed: 77.81%	Importance of image preprocessing demonstrated	[121,122,124,125]
CNN (CystNet hybrid model)	Ultrasound images, Kaggle PCOS dataset	5-fold cross-validation	InceptionV3 + convolutional autoencoder	Dense layer: 96.54% accuracy, RF classifier: 97.75% accuracy	Hybrid architecture with multiple classification approaches	[126]
Deep learning (U-Net + ResNet)	Non-invasive eye imaging (scleral images), *N* = 721 (388 PCOS patients)	Multi-instance learning validation	Sclera segmentation, attention mechanism	AUC: 0.979, accuracy: 92.9%	Novel non-invasive screening using eye imaging	[127]
CNN (PCONet + InceptionV3)	Ultrasound images, Kaggle dataset	Transfer learning validation	Fine-tuned pre-trained models	PCONet: 98.12%, InceptionV3: 96.56%	Custom CNN architecture vs. transfer learning comparison	[128]
**Microbiome analysis**						
Random forest classifier	Stool microbiome profiles, study-specific cohorts	Cross-validation	16S rRNA sequencing, taxonomic profiling	Accuracy: 87.5% (95% CI: 84.2–90.8%), sensitivity: 89.3% (95% CI: 85.6–93.0%), specificity: 85.7% (95% CI: 81.4–90.0%), AUC-ROC: 0.93 (95% CI: 0.90–0.96)	Microbiome-based classification showing promise for non-invasive diagnosis	[22]
Random forest	Gut microbiome and clinical data, multiple cohorts	5-fold cross-validation	Feature selection, diversity metrics	Accuracy: 85% (95% CI: 81–89%), sensitivity: 87% (95% CI: 82–92%), specificity: 83% (95% CI: 78–88%)	Integration of microbiome and clinical parameters	[20]
Random forest	β-diversity with hormonal correlation, study cohorts	Statistical correlation analysis	Microbiome profiling, hormonal measurements	Significant correlation with hyperandrogenism (*p* = 0.0009)	Direct correlation between microbiome and PCOS phenotype	[19]
**Multi-modal** **approaches**						
Machine learning (integrated)	Gut microbiome, BEV-associated miRNAs, clinical parameters, multi-source data integration	Cross-validation	Multi-omics data fusion	Accuracy: 92.0% (CI: 88.9–95.1%), sensitivity: 93.0%, specificity: 91.0%, AUC-ROC: 0.96	Comprehensive multi-omics approach for enhanced accuracy	[33,43,44,47,68]
Deep learning with ensemble	Clinical features and ultrasound images, combined datasets	Cross-validation	Multi-modal feature fusion	SVM: 94.44%, VGG16: 98.29% validation accuracy	Multi-modal data integration approach	[51,129,130]
**Specialized applications**						
Artificial neural networks (Auto-CM)	Clinical characteristics, study-specific	Not specified	Automated feature selection	Performance not specified	Automated clinical decision-making system	[131]
Gradient boosting	Clinical, hormonal, metabolomic data, study cohorts	Cross-validation	Multi-dimensional data integration	AUC-ROC: 0.83	Integration of diverse clinical data types	[118,123,132]
ROC analysis (meta-analysis)	EV-associated miRNAs (miR-29a-5p, miR-320, miR-93), meta-analysis of multiple studies	Multi-study validation	Biomarker standardization	AUC = 0.95 for miR-29a-5p	Meta-analysis approach for biomarker validation	[133,134,135]

Abbreviations: ANNs—Artificial Neural Networks, CNNs—Convolutional Neural Networks, SVMs—Support Vector Machines, RF—Random Forest, XAI—Explainable Artificial Intelligence, SHAP—Shapley Additive Explanations, LIME—Local Interpretable, Model-agnostic Explanations, PCA—Principal Component Analysis, Auto-CM—Automated Clinical Management, U-Net—neural network architecture, ResNet—Residual Networks, VGG16—Visual Geometry Group 16-layer network, TOPSIS—Technique for Order of Preference by Similarity to Ideal Solution, AUC—Area Under the Curve, ROC—Receiver Operating Characteristic, AUC-ROC—Area Under the ROC Curve, CI—Confidence Interval, SMOTEENN—Synthetic Minority Oversampling Technique + Edited Nearest Neighbors.

## Data Availability

No new data were created or analyzed in this study.

## Abbreviations

The following abbreviations are used in this manuscript:

AI	Artificial Intelligence
AI/MLSaMD	Artificial Intelligence and Machine Learning Software as a Medical Device
AMH	Anti-Müllerian Hormone
ANN	Artificial Neural Network
AUC	Area Under the Curve
Auto-CM	Auto-Contractive Map
BCAA	Branched-Chain Amino Acid
BEV	Bacterial Extracellular Vesicle
BMI	Body Mass Index
CNN	Convolutional Neural Network
CREB1	cAMP Responsive Element Binding Protein 1
DOGMA	Dysbiosis Of Gut Microbiota
EV	Extracellular Vesicle
FDA	Food and Drug Administration
FFEV	Follicular Fluid Extracellular Vesicle
FOSL2	FOS Like 2, AP-1 Transcription Factor Subunit
FXR	Farnesoid X Receptor
GDCA	Glycodeoxycholic acid
HIF-1α	Hypoxia Inducible Factor 1 Alpha
HOMA-IR	Homeostatic Model Assessment for Insulin Resistance
IL	Interleukin
IRS-1	Insulin Receptor Substrate 1
LDHA	Lactate Dehydrogenase A
LPS	Lipopolysaccharide
M1/M2	Macrophage phenotypes (M1: pro-inflammatory, M2: anti-inflammatory)
MAPK	Mitogen-Activated Protein Kinase
METTL3	Methyltransferase Like 3
MIF	Macrophage Migration Inhibitory Factor
miRNA	microRNA
MSC-EV	Mesenchymal Stem Cell-Derived Extracellular Vesicle
mTOR	Mammalian Target Of Rapamycin
NF-kB	Nuclear Factor Kappa-Light-Chain-Enhancer of Activated B Cells
NIH	National Institutes of Health
OUT	Operational Taxonomic Unit
PCOS	Polycystic Ovary Syndrome
PI3K-Akt	Phosphoinositide 3-kinase/Protein kinase B pathway
ROC	Receiver-Operating Characteristic
S6K1	Protein S6 Kinase 1
SCFA	Short-Chain Fatty Acid
SCM	Semantic Connectivity Map
sEV	Small Extracellular Vesicle
SIRT1	Sirtuin 1
SMAD5	SMAD Family Member 5
STAT1/STAT3	Signal Transducer and Activator of Transcription 1/3
SVM	Support Vector Machine
TLR2	Toll-Like Receptor 2
TNF-α	Tumor Necrosis Factor alpha
TUDCA	Tauroursodeoxycholic Acid
WNT	Wingless-Related Integration Site
